# CpG Methylation Protects DNA against Ionizing Radiation

**DOI:** 10.1021/acs.jpcb.5c04043

**Published:** 2025-08-20

**Authors:** Leo Sala, Tereza Zápotocká, Jana Šáchová, Václav Olšanský, David Chvátil, François Chevalier, Violaine Vizcaino, Alain Méry, Jaroslav Kočišek

**Affiliations:** † Department of Dynamics of Molecules and Clusters, 86875 J. Heyrovský Institute of Physical Chemistry of the CAS, Dolejškova 3, 182 23 Prague, Czech Republic; ‡ Laboratory of Genomics and Bioinformatics, 89219 Institute of Molecular Genetics of the CAS, Vídeňská 1083, 142 20 Prague, Czech Republic; § 226313 Nuclear Physics Institute of the CAS, Řež 130, 250 68 Řež, Czech Republic; ∥ Normandie Univ, ENSICAEN, UNICAEN, CEA, CNRS, CIMAP, Boulevard Henri Becquerel, BP 5133, 140 70 Caen cedex 5, France

## Abstract

Methylation of DNA CpG domains in cellular DNA is a key mechanism of epigenetic regulation. Disruptions in the processes maintaining DNA methylation can lead to diseases like cancer. The radiation response of certain cancer cells may be affected by their DNA methylation levels, which may have consequences in their response to radiotherapy. In this work, we utilized DNA origami nanotechnology to examine whether DNA methylation impacts DNA response to ionizing radiation in solution before biological processes come into play. Our findings reveal that a protective effect is achieved with just a few methylated CpG adducts. Both low-LET (electron) and high-LET (carbon ion) irradiation show a reduced lesion count in methylated DNA, as indicated by qPCR results. AFM single-molecule observations using DNA origami nanoframes suggest fewer double-strand breaks in methylated DNA after carbon ion irradiation. This radioprotective effect may contribute to the differential radiation response of cellular DNA and should be considered when predicting and evaluating DNA radiation damage yields.

## Graphical abstract

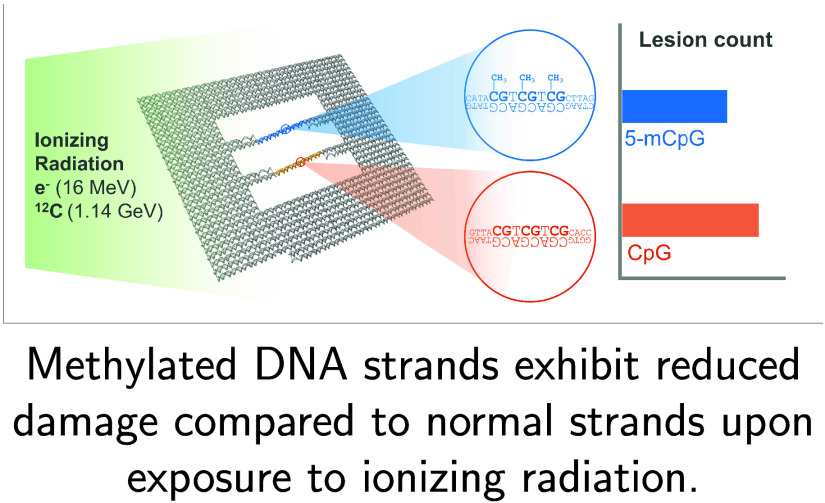

## Introduction

DNA methylation has been identified as one of the key epigenetic factors shaping gene expression. This intricate process occurs predominantly within the CpG regions of the genome *via* the conversion of cytosine nucleobases into 5-methylcytosine (5mC). During growth and development, the DNA methylation patterns of organisms evolve, adapting to various environmental and physiological signals.^1^ However, this transformation comes with a trade-off: 5mC is prone to deamination, producing thymine and causing G-T base mismatches that can activate DNA repair mechanisms.^2^ The ability of certain organisms, especially mammals, to endure the mutagenic potential of 5mC suggests the existence of sophisticated demethylation pathways. Typically, this process involves the hydroxylation of 5mC to 5-hydroxymethylcytosine (5hmC) by ten-11 translocation (TET) methylcytosine dioxygenases, followed by the removal of oxidized residues by thymine DNA glycosylases, effectively restoring cytosine.^1,2^

Disruptions to the delicate balance of DNA methylation can have profound consequences. Hypermethylation may silence critical genes, such as tumor suppressors^3^ or genes associated with DNA repair,^4^ while hypomethylation can destabilize the genome and activate genes that are otherwise dysregulated.^5^ These imbalances are often linked to a variety of genetic and oncogenic disorders, most notably cancer.^6–9^

Radiotherapy, one of the most effective cancer treatment modalities today, has been shown to influence DNA methylation patterns. Activating DNA repair mechanisms can lead to aberrant methylation changes.^10,11^ In various *in vitro* and *in vivo* explorations, ionizing radiation has been shown to induce hyper- or hypomethylation, depending on a complex interplay of physicochemical and biological factors related to the cell line or mouse model types,^12^ linear energy transfer (LET),^13,14^ and tumor environment.^15^ Additionally, the baseline methylation levels of cells may also influence their radiosensitivity.^7^ The intricate matrix of influences arising in the biological stage of radiation interaction with living tissue complicates the prediction of therapeutic outcomes and consequently, the rational design of radiotherapeutic strategies. In the present work, we performed *in singulo* experiments on isolated and precisely defined DNA sequences to at least, identify and isolate the effect of 5mC during the primary, physicochemical stage of the interaction.

While the biological response to radiation is frequently attributed to disturbances in cellular mechanisms, such as influencing DNA repair pathways, mechanisms affecting radiation response already start to occur at the physicochemical level, during the primary interaction of ionizing radiation with DNA and its close environment.^16^ The sensitivity of chemically modified DNA to radiation often manifests at the primary structural level.^17,18^ For instance, studies on ultraviolet (UV)-induced damage have shown a reduced yield of photoproducts in DNA oligomers containing methylated cytosine.^19^

In this work, we investigate the innate radiation response of methylated DNA by utilizing DNA origami nanoplatforms. Through this method, precise quantification of radiation-induced damage to specific DNA sequences *in singulo* is possible.^20,21^ Adapting the DNA origami nanoframe design by Endo et al.,^22^ we secured two DNA sequences within the inner aperture of the nanoframe, enabling a direct comparison between them. While Endo et al. demonstrated how helical tension in methylated DNA can regulate methyltransferase activity,^22^ our focus is geared toward investigating radiation damage yields between methylated and unmethylated DNA strands. Employing DNA origami nanoframes ensures that both strands are exposed to the same radiation dose and environmental conditions, allowing for a more accurate comparison of strand break yields. The method also facilitates sequence control to zoom into specific DNA segments which are often difficult to achieve with conventional plasmid-based approaches used in studying DNA radiation damage.^23^ The exceptional stability of DNA origami nanostructures under various types of ionizing radiation, spanning low to high LET regimes, makes them ideal substrates for irradiation experiments.^24,25^ Here we compare the effects of low LET radiation represented by 16 MeV electrons and high LET radiation represented by 1.14 GeV (95 MeV u^–1^) ^12^C ions.

The methodology developed in our group^23^ consists of the synthesis of DNA nanoframes with two parallel DNA sequences in the inner aperture (shown in Figure 1), their irradiation in buffer solutions, and analysis of strand breaks in the parallel DNA sequences. High-resolution imaging of multiple DNA origami nanoframes was achieved using atomic force microscopy (AFM) to visualize individual strand breaks. However, single-strand breaks could not be resolved as they are within AFM lateral resolution limits. To address this, Quantitative Polymerase Chain Reaction (qPCR) was employed to complement the AFM findings, providing quantitative data on the total lesions sustained by the DNA strands following exposure to ionizing radiation.

**Figure 1:**
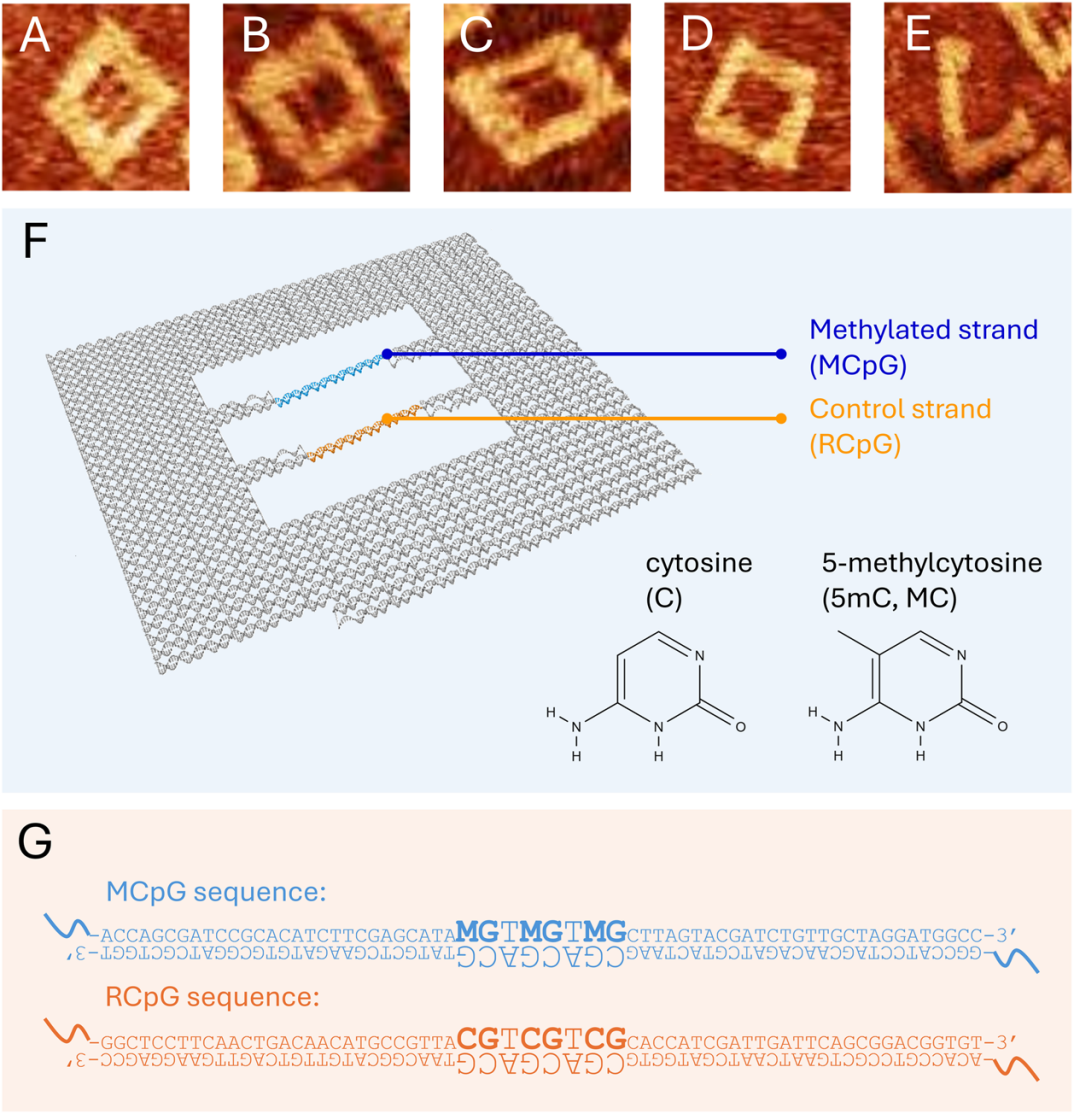
Schematic of the DNA origami nanostructure design, AFM characterization, and sequences of interest. Representative AFM images of DNA origami nanoframes with DNA motifs of interest at various stages of damage are shown in the top lane: intact (A), top methylated strand (MCpG) broken (B), bottom control strand (RCpG) broken (C), both motifs broken (D), and nanoframe fragmentation at high doses (E). A sketch of the DNA origami nanoframe is displayed in (F) indicating the strand placements of the DNA motifs studied with their respective primary sequences shown in (G) highlighting the precise position of 5mC (denoted by the letter M). The chemical structure of cytosine and 5mC is also shown in F for reference.

## Experimental Section

### DNA Origami Synthesis

A 100 μL reaction mixture containing 6 nM M13mp18 ssDNA scaffold (Tilibit Nanosystems), 60 nM DNA origami nanoframe staple mix (Metabion), and 60 nM of the desired DNA motifs (MCpG and RCpG, Metabion) was assembled in 1× TAE with 12.5 mM MgCl_2_ (Sigma-Aldrich). The sequences of the duplex strands used are listed in Table 1. The mixture was annealed to 90 °C for 5 min and slowly cooled to room temperature at a rate of −7 °C min^–1^ in a heating block with temperature control.^24^ The resulting mixture was then filtered through 100 kDa MWCO Amicon filters (Millipore) three times to remove excess staples, adding 400 μL of 0.25× TAE with 12.5 mM MgCl_2_ (irradiation buffer) each time. The concentration of the filtered mixtures was checked by measuring the UV absorbance at 260 nm using a Denovix DS-11 FX+ spectrophotometer. This synthesis typically results in 50 μL of 12–15 nM of DNA origami nanoframe solutions which were then diluted or reproduced as required.

**Table 1:** Sequences of the DNA Motifs Investigated

label	position	description	sequence (5′-3′)
MCpG	top duplex	central methylated CpG domains	CTGTAGCTCATCATGTACCAGCGATCCGCACATC-TTCGAGCATA**MG**T**MG**T**MG**CTTAGTACGATCTG-TTGCTAGGATGGCC^a^
		complement	GACGGGAGAATTAACTGGCCATCCTAGCAACAGATCGTACTAAGCGACGACGTATGCTCGAAGATGTGCGGATCGCTGGT
RCpG	bottom duplex	central CpG domains	TTGCCTGAGAGTCTGGGGCTCCTTCAACTGACAACATGCCGTTA**CG**T**CG**T**CG**CACCATCGATTGATTCAGCGGACGGTGT
		complement	CGACAATAAACAACATACACCGTCCGCTGAATCAATCGATGGTGCGACGACGTAACGGCATGTTGTCAGTTGAAGGAGCC

a M = 5-methylcytosine.

### Electron Irradiation

The electron irradiation experiments were performed at the Mictrotron MT25 accelerator of the Nuclear Physics Institute of the Czech Academy of Sciences. The details of the accelerator are described elsewhere.^26^ It produces monoenergetic electron beams of energies between 6 and 25 MeV with only tens of keV in energy dispersion. DNA nanoframe solutions (6 nM, 100 μL) in 1.5 mL Eppendorf tubes were placed onto a dedicated rotating sample holder. They were then irradiated with 16 MeV electrons. The sample holder was placed 45 cm from the beam exit where the dose rate was measured prior to irradiation using a TN34045 ionizing chamber (PTW Freiburg) and by a Keithley 617 programmable electrometer. The dose rates were varied between 10–20 Gy s^–1^ depending on the absorbed dose. At the front and back ends of the sample holder the dose rates only varied by ±10%. Significant sample heating was not observed, although a cooling fan was placed near the sample holder to help maintain the ambient temperature.

### Carbon Ion Irradiation

The ^12^C ion irradiation experiments were done at the IRABAT (IRradiation À BAsse Temperature) beamline of the GANIL (Grand Accélérateur National d’Ions Lourds) facility in Caen, France. The beamline is equipped with a dedicated dosimetry and a beam sweeping device that can uniformly irradiate a typical irradiation field of 5 cm × 5 cm with an ion fluence accuracy of ∼ 5%, horizontal frequency of 400 Hz, and vertical frequency of 4 Hz.^27^

A 95 MeV u^–1 12^C ion (1.14 GeV) beam was generated to irradiate the DNA nanoframe samples (6 nM, 100 μL) inside 1.5 mL Eppendorf tubes placed on a dedicated sample holder. The maximum flux was set to 1.3 × 10^8^ ions cm^–2^ s^–1^ equivalent to 5.3 Gy s^–1^. The ion flux was deduced from the measurement of the beam intensity using a detector based on the secondary electron emission from a thin Fe foil placed inside the IRABAT vacuum chamber, serving as an online flux monitor during sample irradiation. A Faraday cup was used to calibrate this detector before irradiation.

### Quantitative Polymerase Chain Reaction (qPCR)

The quantity of remaining intact duplex strands after irradiation was assessed using a SYBR Green-based quantitative PCR assay (SYBR Green I Master Kit, Roche Applied Science) performed on the LightCycler 480 system. Two microliters of either control or irradiated DNA nanoframe solutions were mixed with a customized primer solution to achieve a final primer concentration of 1 μM in a reaction volume of 5 μL. The cycling program began with an initial denaturation step at 95 °C for 5 min, followed by 40 cycles of denaturation at 95 °C for 15 s, annealing at 55 °C for 30 s, and extension at 72 °C for 30 s, capped with an additional 10-s extension at 72 °C. To ensure the specificity of amplification and rule out nonspecific products or primer-dimer formation, melting curve analyses were conducted following the amplification step. Several primer sets of varying lengths were tested, and those with the highest efficiency and specificity were selected (listed in Table 2). Primer efficiencies were determined using standard curves generated from a ∼5 μM DNA stock solution containing the target amplicon, serially diluted to concentrations of 10*^n^* x, where *n* = 2–5.

**Table 2:** Primers Used for RT-PCR

amplicon	forward primer sequence (5′-3′)	reverse primer sequence (5′-3′)	efficiency
MCpG	ACCAGCGATCCGCACATCTTC	GCCATCCTAGCAACAGATCG	94%
RCpG	TTGCCTGAGAGTCTGGGGCT	CCGTCCGCTGAATCAATCG	83%

The number of total lesions (μ_TL_) on the irradiated DNA motifs was evaluated from the linear fit of the dose (*D*) dependence (eq 1) of the qPCR fluorescence threshold values (*C*_p_), assuming a simple exponential dose dependence of the concentration of the remaining intact DNA motifs. The details of which are described elsewhere.^23^ This equation also takes into account the primer efficiency (*E*). *C*_p,N_0__ denotes the fluorescence threshold value of the unirradiated control sample. The average *C*_p_ ± SD value for each absorbed dose was obtained from measured *C*_p_ values of four replicates.

1 $$C_{p}=\frac{\mu_{\mathrm{TL}}D}{\ln\left(1+E\right)}-C_{p,N_{0}}$$

### Atomic Force Microscopy

Si substrates (∼0.5 cm × 0.5 cm) were plasma cleaned for 10–20 s using the Roplass RPS40+ plasma cleaner. A 1 μL drop of 6 nM DNA origami nanoframe solution is placed in the middle of the substrate with an additional 15 μL drop of 10xTAE solution with 125 mM MgCl_2_. The sample was left to incubate for 1 h over an ethanol bath. After incubation, the sample was washed with 1 mL of 50% ethanol solution and then dried with a stream of N_2_ gas. Imaging of the dried samples was done in air using the Dimension Icon AFM (Bruker) utilizing PeakForce Tapping Technology and ScanAsyst probes (40 kHz, 0.4 N m^–1^). The obtained images were then flattened using the Gwyddion software and the number of double strand breaks on each nanoframe in the images were marked and evaluated using the Cell Counter plug-in in the ImageJ software.

The double strand break yields (μ_DSB_) were approximated for the quasi-linear range of the dose dependence curve for the counted double strand breaks (*N*_DSB_). The approximation is shown in eq 2 and described in detail elsewhere.^23^ μ_DSB_ denotes the number of double strand breaks already observed from the unirradiated control samples. An average of ∼1000 nanoframes per absorbed dose was counted, which was attained by collecting and analyzing 4–12 images per absorbed dose depending on the surface coverage.

2 $$N_{\mathrm{DSB}}\approx \mu_{\mathrm{DSB}}D+N_{{\mathrm{DSB}}_{0}}$$

## Results and Discussion

### Low-LET Irradiation

Electrons with energies of 16 MeV were used to model low-LET irradiation effects. The total lesions per dose was evaluated from the RT-PCR threshold values (*C*_p_) assuming a simple exponential dose-dependence (see Experimental Section). The raw *C*_p_ values measured for each absorbed dose are plotted in Figure 2A for both the methylated and unmethylated strands on the same DNA origami nanoframe. This method does not differentiate between the types of lesions that may impede PCR propagation; hence, the slope reflects the increase in the total number of lesions (Methods section, eq 1). The total lesions comprise all breaks (single and double strand) as well as any damage to the strands that hinders PCR amplification, including base loss or base damages that result in mispairing or double-helix distortions that interfere with polymerase binding. The estimated total lesion counts are listed in Table 3 and compared in Figure 3A. It appears that there are more pronounced lesions on the unmethylated strand compared to the methylated strand. The total lesion yield is decreased by a factor of 0.77 ± 0.09 when the strand is methylated showing some level of radioprotection.

**Table 3:** Estimated Strand Break Yields and Radio-Enhancement Factors (EF) on Methylated (MCpG) and Unmethylated Strands (RCpG) after Irradiation^a^

DNA strand	radiation type	μ_TL_ ± SD (total lesions kGy^–1^)	EF_TL_	μ_DSB_ ± SD (DSB kGy^–1^)	EF_DSB_
MCpG	electrons	0.16 ± 0.02	0.77 ± 0.09	0.065 ± 0.003	0.98 ± 0.05
RCpG	electrons	0.21 ± 0.01		0.066 ± 0.002	
MCpG	carbon ions	0.15 ± 0.02	0.74 ± 0.10	0.026 ± 0.004	0.74 ± 0.13
RCpG	carbon ions	0.20 ± 0.02		0.035 ± 0.003	

a EF is the ratio of the lesion- or strand break yield of the modified strand (MCpG) to the control (RCpG).

**Figure 2:**
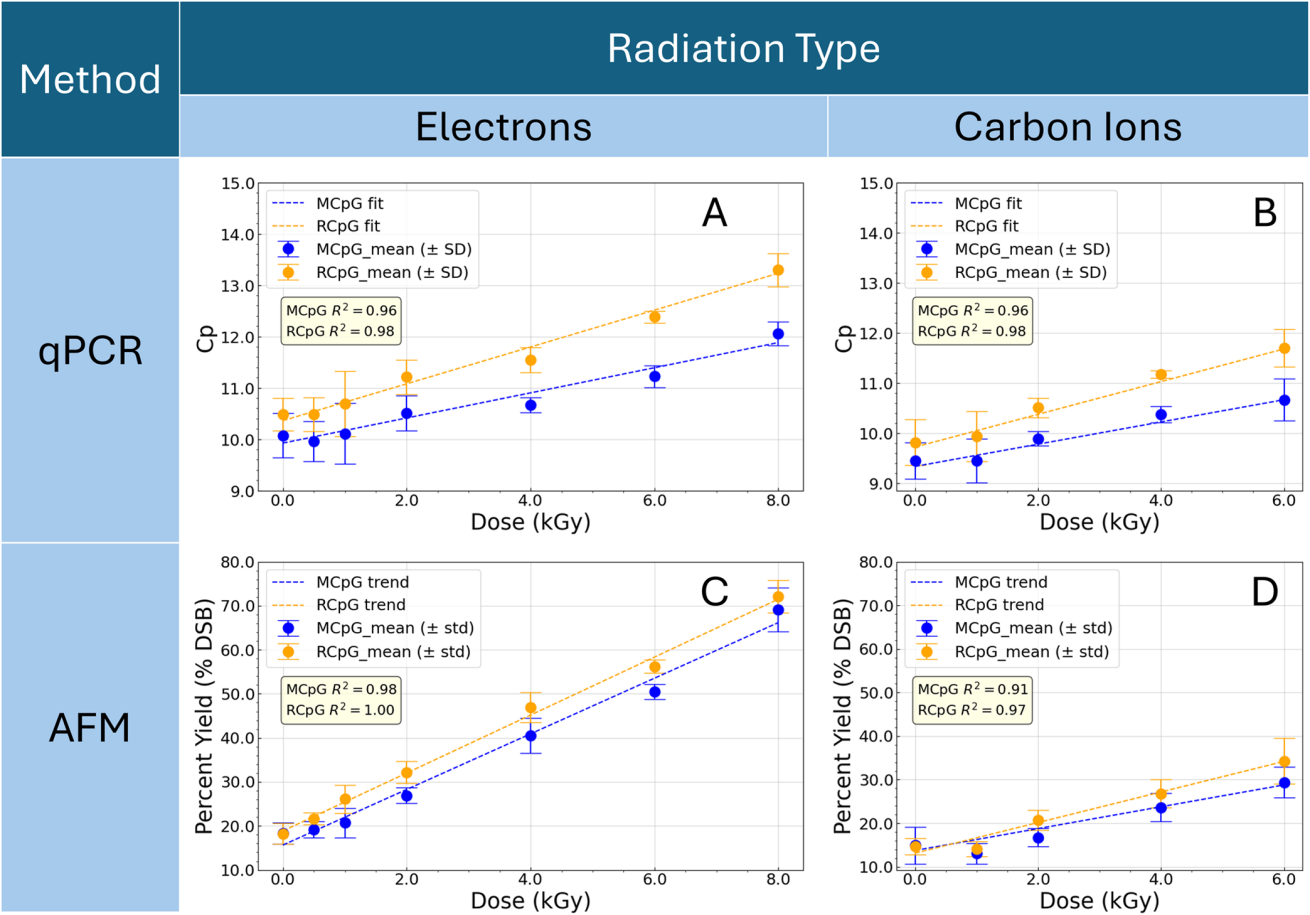
Absorbed dose-dependent damage to the sequences of interest evaluated using qPCR and AFM. Top row: *C*_p_ values (Mean ± SD, *n* = 4) from qPCR measurements *vs* absorbed dose after electron (A) and carbon ion (B) irradiation of DNA nanoframes containing DNA motifs with methylated CpG (blue) and the accompanying RCpG control strand (orange). Bottom row: AFM counts of % DSB (Mean ± SD, *n* = 4–12) *vs* absorbed dose curves after electron (C) and carbon ion (D) irradiation. Linear fits were performed on each curve based on eqs 1 and 2 for data obtained from qPCR and AFM, respectively.

**Figure 3:**
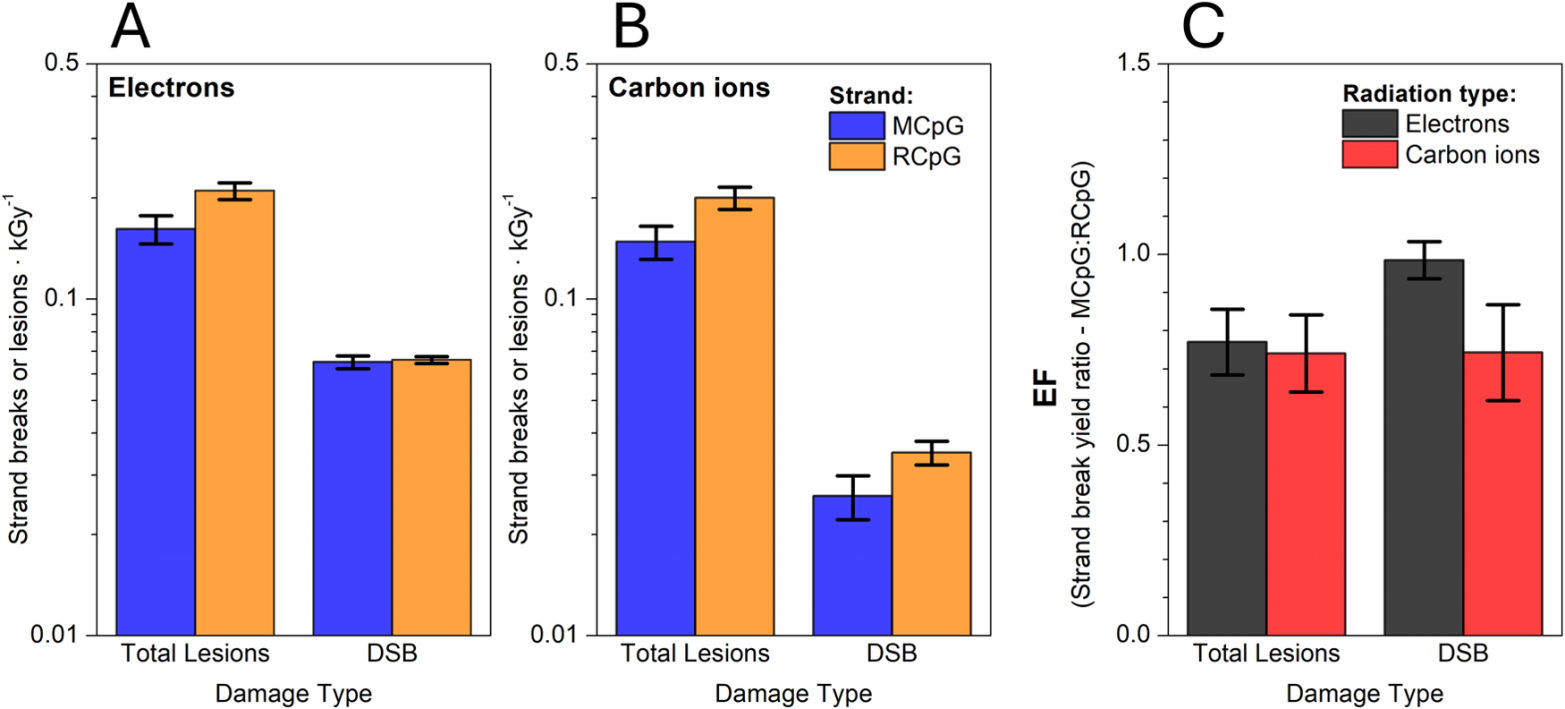
Comparison of total lesion and DSB yields between DNA motifs with methylated CpG adducts (MCpG, blue bars) and the accompanying control, unmethylated strand (RCpG, orange bars) during electron (A) and carbon ion (B) irradiation. Enhancement ratios (EF) comparing the lesion/DSB yields between the methylated and unmethylated strands are shown in (C) for electron (black bars) and carbon ion (red bars) irradiation. The values of the damage yields are listed in Table 3.

Double strand break yields (DSB) in Table 3 were estimated from the slope of the absorbed dose–response curves (Figure 2C) of DSB counts from AFM images. A comparison of the yields is shown in Figure 3A. Unlike the total lesion count, the DSB yield is barely changed by the introduction of methylated domains (EF = 0.98 ± 0.05). Comparing the DSB values to the total lesion count, the proportion of DSB among the total lesions in the methylated strand is higher than in the unmethylated strand. Although there is a reduction in the total lesion count when the strand is methylated, the relative probability of such modified DNA to form double strand breaks is higher.

### High-LET Irradiation

For high-LET irradiation, 1.14 GeV ^12^C ions were used. The qPCR methodology was the same as that used for the electron-irradiated samples. The plot of the dose-dependence of C_p_ values is shown in Figure 2B for both the methylated and unmethylated strands irradiated with ^12^C ions. The estimates of the total lesion yields are listed in Table 3 with a graphical comparison in Figure 3B. A similar protective effect is observed as in the case of low-LET based on the total lesion yields which are decreased by a factor of 0.74 ± 0.10 when the strands are methylated.

Double-strand break yields evaluated by AFM obtained from the slope of the dose-dependence curve in Figure 2D are tabulated in Table 3 with a comparison between the damages to the methylated and unmethylated strand illustrated in Figure 3B. There also appears to be some protective effect which decreases the DSB yield by a factor of 0.74 ± 0.13. The high variability in these results might have been caused by the already significant damage to the nanoframes at higher absorbed doses, *i.e.*, ∼80% of the nanoframes were already damaged (Figure S1). High-LET could lead to nanoscale damages^28^ that challenge the structural integrity of the DNA origami nanoframes. This fragmentation may lead to an underestimation of the strand break damage, since there is a higher probability that the completely damaged frame will bear damaged DNA strands than it will bear undamaged DNA. A similar effect has been observed on ion beam irradiated plasmid DNA where strand break yields were also underestimated as a result of fragmentation.^29^ Even though this effect could have influenced the total number of strand breaks evaluated, it has no effect on the observed ratios between the parallel methylated and unmethylated strands. We can conclude that although methylation conveys some level of radioprotection for all types of lesions, it may not have influenced the proportion of DSBs incurred.

### Impact of LET

Before comparing the results between the low- and high-LET irradiation experiments, we should stress here that our single-molecule approach is focused on the evaluation of the relative damage between two parallel double-strand DNA segments inside the nanoframes. This number is not influenced heavily by systematic errors such as different dose rates, dosimetry, experiment configurations or variations in scavenger concentrations.^30^ The following discussion comparing the apparent strand break yields between high- and low-LET, on the other hand, could be influenced by the above-mentioned factors and needs to be addressed with careful consideration.

For high-LET radiation, both direct and indirect effects of radiation are distributed within the vicinity of the particle track. In contrast, low-LET radiation results in a more dispersed distribution of ionization events, leading to scattered damages.^31^ Consequently, the high LET radiation generally induces more clustered damages,^32,33^ which may lead to a higher yield of double-strand breaks.^34^ From this point of view, our results considering the differences between electrons and carbon ions may be unexpected, since in our case, the low-LET electron irradiation results in a higher relative yield of DSBs than carbon ion irradiation. This behavior can be, however, well explained by the process dominating the creation of double-strand breaks in solution, which involves OH radical attack.^35,36^ To estimate the OH radical yield, we evaluated the number of OH radicals in the radiolysed solutions using UV–vis by measuring the absorbance of tris–OH products (adapted from^37,38^ ). In the present case, the OH radical yields in high-LET carbon ions are lower by about 2.2 times (Figure S2) compared to low-LET electrons. This ratio is in good agreement with the observed ratio of 2.5 ± 0.4 and 1.9 ± 0.2 between DSBs induced by the electrons to carbon ions in MCpG and RCpG strands, respectively. The higher damaging effect of electron irradiation in the present case is also well supported by gel electrophoresis results from denatured samples, which also indicate greater cumulative damage to both CpG strands in electron irradiation compared to carbon ion irradiation (Figure S3–C in the Supporting Information).

However, these observations do not correlate with the evaluated dose dependencies of the total number of lesions evaluated by PCR. Comparing the numbers in the third column of Table 3, we can see that the number of lesions for low-LET and high-LET are identical. There are three possible explanations for this. The first is that while in the case of electrons, the total number of lesions is primarily caused by OH radical damage, the situation is different for carbon ions. It is widely recognized that with increasing LET, the indirect effects of ionizing radiation decrease;^39^ however, this does not explain well the observed correlation of double-strand break and OH radical yields. Such behavior was observed also in previous studies of plasmid DNA, showing a strong correlation of DSBs with OH radical yield, while practically no correlation was observed with the amount of clustered damages.^35^ Since clustered damages become dominant for high LET, we believe these differences in the damage mechanisms may cause a higher yield of the total lesion in the case of carbon ions than expected based on the relative OH radical yield. This effect is further amplified by the used methodology where clustered damages occurring on the individual strands of the same duplex but not resulting in double-strand breaks are counted in the total number of lesions, but not detected as DSBs using AFM.

The second possible explanation is related to dose rate effects. In the present experiment, we chose to work at a higher dose rate for electrons, to minimize OH recombination effects and ensure a comparable chemical environment to carbon ion irradiation (Figure S2 in the Supporting Information). Under these conditions, the high electron flux may have led to more clustered damages and higher conversion efficiency of SSBs to DSBs. A similar effect has been observed for XUV irradiated plasmid DNA.^40^ This is supported also by a comparison with our previous study of modified DNA strands on DNA origami nanoframes.^23^ Although the sequences in that study differ from the current work, making a direct comparison complicated, the ratio of SSBs to DSBs incurred after electron irradiation at a lower dose rate was higher than in the present study. In this case, the SSB to DSB ratio is approximately ∼3 for the control strands, while it was around ∼15–20 in the previous work (Table 1 in ref 23). Increased conversion ratios with higher dose rates have also been observed for plasmids irradiated with 16 MeV electrons.^41^ Meanwhile, the conversion ratio is ∼6 for carbon ion irradiation in the present work.

Finally, the absolute values of the strand breaks may be influenced by many environmental factors such as oxygen levels^42^ or the high magnesium environment needed to stabilize DNA origami nanostructures.^43^ These may result in a complex radiation chemistry. The oxidation of 5mC results in the formation of various products,^44^ some of which exert a stabilizing effect, while others may promote strand breakage.^45,46^ For example, HPLC measurements have shown that in gamma-irradiated DNA, 5-methylcytosine (5mC) is more likely to react with hydroxyl radicals than unmodified cytosine. However, the major oxidation products of 5mC, such as 5-formylcytosine (5fC) and 5-hydroxymethylcytosine (5hmC), are chemically stable and do not immediately lead to backbone cleavage.^44^ The presence of 5mC also reduces the yield of 8-oxoguanine (8-oxoG), a lesion often associated with strand breaks due to its highly reactive further oxidation products. Quantum chemical calculations on isolated nucleotides and on di- or trinucleotide sequences suggest that the ionization potential is slightly lowered in the presence of 5mC, potentially allowing it to act as a sacrificial nucleobase that forms more stable oxidation products.^45,46^ However, such chemical modifications may affect base pairing fidelity, which could help explain why only subtle differences in DSB yields are observed between methylated and unmethylated DNA strands. A more detailed analysis of the radiolytic products of DNA is needed to test these hypotheses, but this remains challenging due to their low concentrations.

## Conclusions

This work demonstrated the use of DNA origami nanostructures to evaluate the effect of methylated CpG domains on radiation-induced DNA strand damage. Regardless of LET, the total strand break yields on strands with methylated CpG domains were reduced, indicating a protective effect against both direct ionizing radiation and secondary reactive species produced in the medium. However, protection against double-strand breaks (DSBs) was less pronounced with low-LET irradiation, and methylation actually increased the proportion of DSBs. Based on measurements of OH radical yields, we hypothesize that this effect could be associated with the modification of the reactivity of the methylated strands with OH radicals. Profiling of radiolysis products from methylated DNA–something not assessable with the current method–could be conducted to better understand the mechanisms behind the observed radioprotection. For high-LET irradiation, methylation did not increase the proportion of DSBs but suggested a global protective effect. This effect may also contribute to the observed differential radiosensitivity of genomic DNA, potentially in conjunction with protective effects associated with secondary structures.^47^ Additionally, the strand break yields derived from this study raise several important questions regarding dose rates and environmental factors affecting radiation-induced damage, which could be explored further in future research.

## Supplementary Material



## Notes

Dose-dependence of nanostructure damage, dose-dependence of tris-OH production measured by UV–vis spectrophotometry, agarose gel electrophoresis of irradiated samples (PDF)
