# Supplementary material for: CpG Methylation Protects DNA against Ionizing Radiation
Source: J Phys Chem B. 2025 Aug 20;129(35):8880–7. doi: 10.1021/acs.jpcb.5c04043 (PMC12415931; doi:10.1021/acs.jpcb.5c04043)
Supplement: Supplementary file 1 [file jp5c04043_si_001.pdf]

Supporting information: CpG methylation protects DNA against ionizing radiation

Leo Sala,[*,†] Tereza Zápotocká,[†] Jana Šáchová,[‡] Václav Olšanský,[†,¶] David Chvátil,[¶] François Chevalier,[§] Violaine Vizcaino,[§] Alain Méry,[§] and Jaroslav Kočišek[*,†]

†*Department of Dynamics of Molecules and Clusters, J. Heyrovský Institute of Physical Chemistry of the CAS, Dolejškova 3, 182 23, Prague, Czech Republic*

‡*Laboratory of Genomics and Bioinformatics, Institute of Molecular Genetics of the CAS, Vídeňská 1083, 142 20, Prague, Czech Republic*

¶*Nuclear Physics Institute of the CAS, Řež 130, 250 68, Řež, Czech Republic*

§*Normandie Univ, ENSICAEN, UNICAEN, CEA, CNRS, CIMAP, Boulevard Henri Becquerel, BP 5133, 140 70, Caen cedex 5, France*

E-mail: leo.sala@jh-inst.cas.cz; jaroslav.kocisek@jh-inst.cas.cz

---

List of Figures

DNA origami nanoframe damage count S2

Relative hydroxyl radical yields from UV absorbance of tris-OH products S3

AGE analysis of denatured samples S4

Radiation damage to DNA origami nanoframes

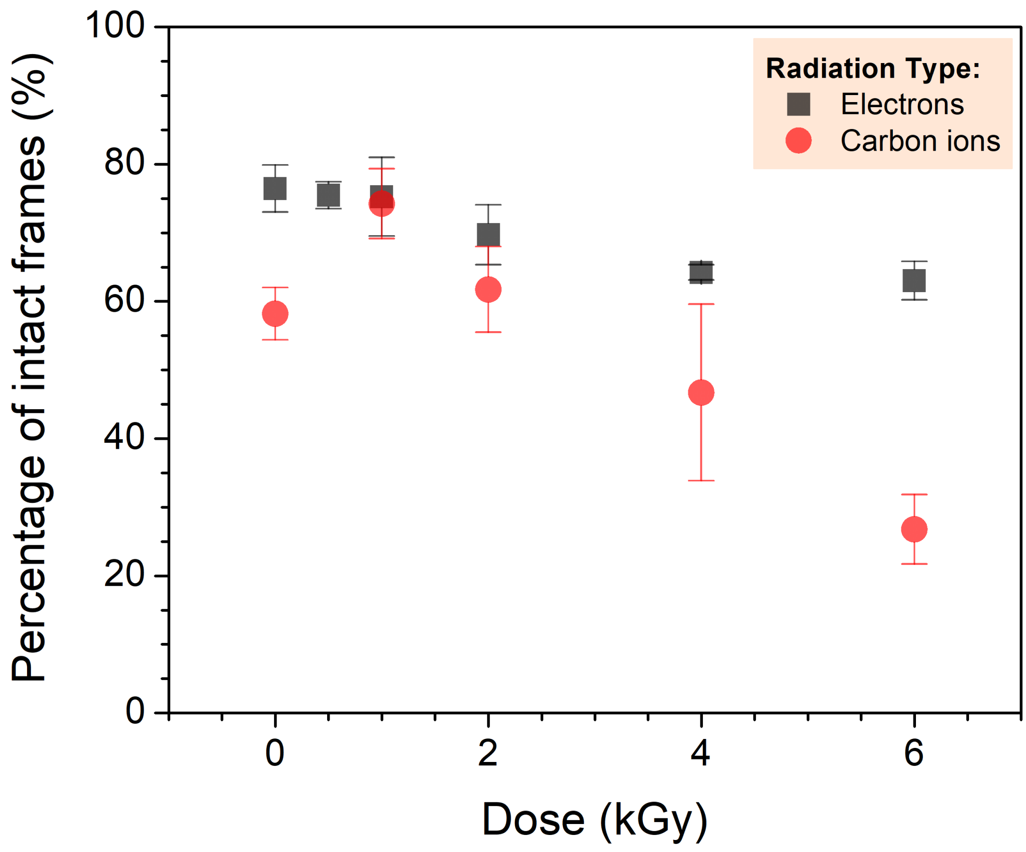

Figure S1: Percentage of intact frames left after exposure to various absorbed doses of electrons (black squares) and carbon ions (red circles).

Relative OH radical yields

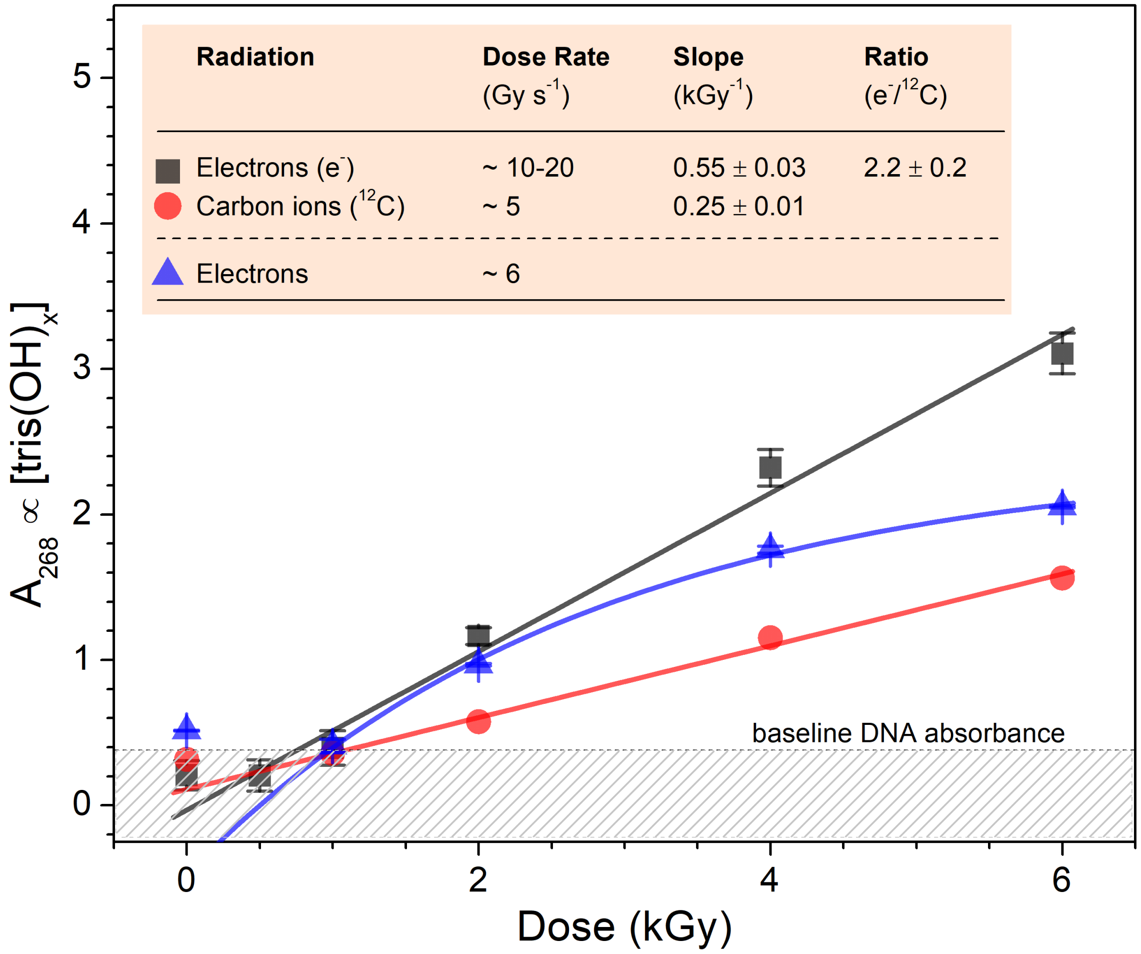

Figure S2: UV absorbance at 268 nm of DNA origami solutions in tris-containing buffer ([tris] = 10 mM) vs. absorbed dose after irradiation with electrons (black squares) and carbon ions (red circles) and the approximate linear fits to estimate the average radical yield ratio between the two irradiation conditions. Lower dose rate electron irradiation was also tested (blue triangles) which resulted in a non-linear profile of tris-OH yields vs. dose suggesting that the rate of OH radical generation is slower than the rate of OH recombination. The former two conditions were therefore adapted for the DNA irradiation experiments to have comparable conditions between electron and carbon ion irradiation, respectively.

AGE analysis of denatured samples

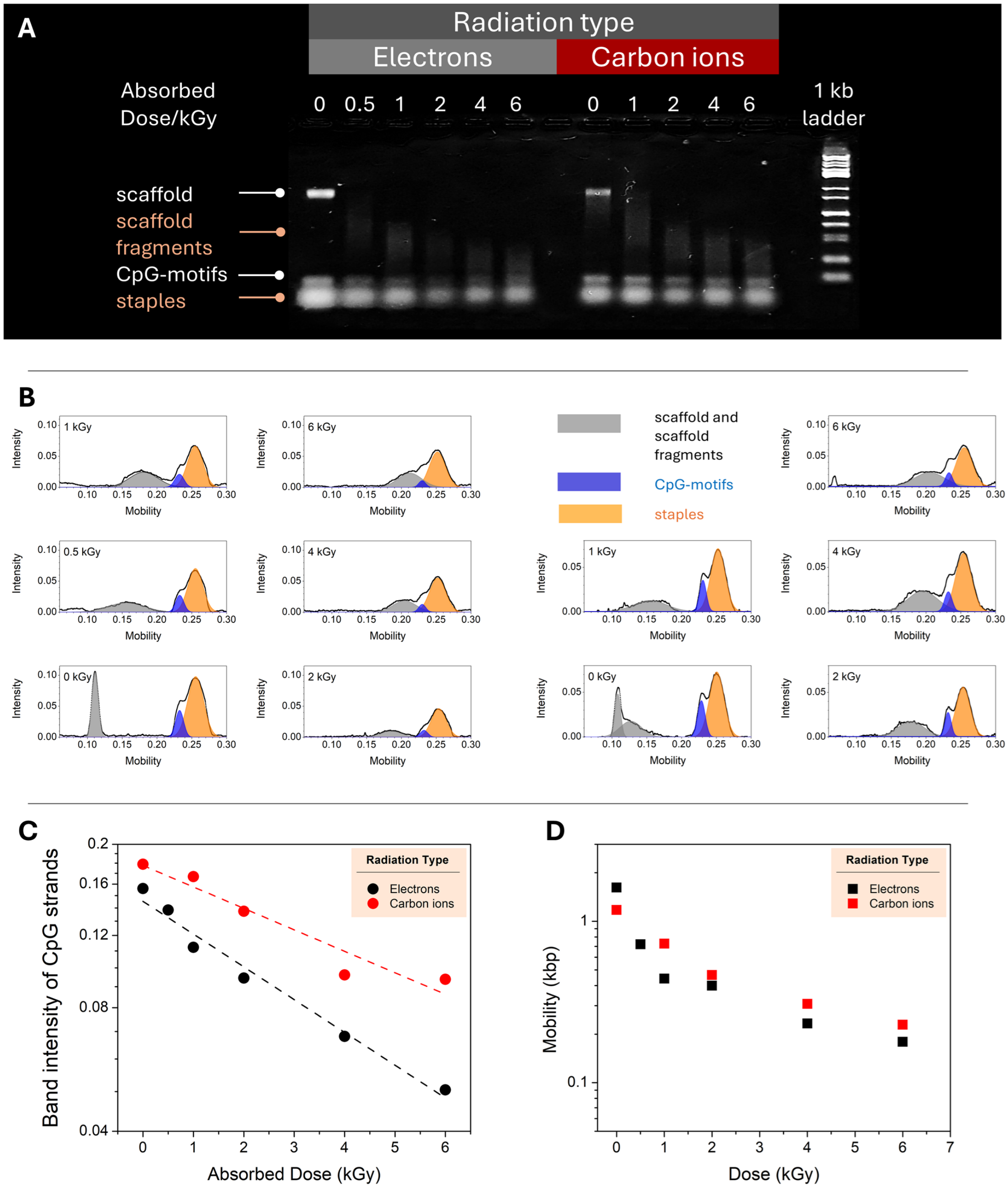

Figure S3: AGE analysis of denatured samples. Raw gel image (A) of lanes loaded with controls, electron-, and carbon ion-irradiated samples. The extracted profiles of the lanes are shown in B with gaussian fits corresponding to the various bands associated to the components of the denatured sample. The dose dependent evolution of the estimated band intensity containing both the methylated and unmethylated strands is plotted in C while D shows the relative mobility of the DNA origami scaffold/scaffold fragments approximated from the 1 kb ladder mass steps.
